# Genetic Characterization of Venezuelan Equine Encephalitis Virus from Bolivia, Ecuador and Peru: Identification of a New Subtype ID Lineage

**DOI:** 10.1371/journal.pntd.0000514

**Published:** 2009-09-15

**Authors:** Patricia V. Aguilar, A. Paige Adams, Victor Suárez, Luis Beingolea, Jorge Vargas, Stephen Manock, Juan Freire, Willan R. Espinoza, Vidal Felices, Ana Diaz, Xiaodong Liang, Yelin Roca, Scott C. Weaver, Tadeusz J. Kochel

**Affiliations:** 1 Naval Medical Research Center Detachment, Lima, Peru; 2 Department of Pathology and Center for Tropical Diseases, University of Texas Medical Branch, Galveston, Texas, United States of America; 3 National Institute of Health, Lima, Peru; 4 Office of Epidemiology, Lima, Peru; 5 Centro de Enfermedades Tropicales, Santa Cruz, Bolivia; 6 Hospital Vozandes del Oriente, Shell, Ecuador; 7 Hospital de la IV División de Amazonas, Puyo, Ecuador; 8 Facultad de Ciencias, Universidad Nacional Agraria La Molina, Lima, Peru; Yale School of Public Health, United States of America

## Abstract

Venezuelan equine encephalitis virus (VEEV) has been responsible for hundreds of thousands of human and equine cases of severe disease in the Americas. A passive surveillance study was conducted in Peru, Bolivia and Ecuador to determine the arboviral etiology of febrile illness. Patients with suspected viral-associated, acute, undifferentiated febrile illness of <7 days duration were enrolled in the study and blood samples were obtained from each patient and assayed by virus isolation. Demographic and clinical information from each patient was also obtained at the time of voluntary enrollment. In 2005–2007, cases of Venezuelan equine encephalitis (VEE) were diagnosed for the first time in residents of Bolivia; the patients did not report traveling, suggesting endemic circulation of VEEV in Bolivia. In 2001 and 2003, VEE cases were also identified in Ecuador. Since 1993, VEEV has been continuously isolated from patients in Loreto, Peru, and more recently (2005), in Madre de Dios, Peru. We performed phylogenetic analyses with VEEV from Bolivia, Ecuador and Peru and compared their relationships to strains from other parts of South America. We found that VEEV subtype ID Panama/Peru genotype is the predominant one circulating in Peru. We also demonstrated that VEEV subtype ID strains circulating in Ecuador belong to the Colombia/Venezuela genotype and VEEV from Madre de Dios, Peru and Cochabamba, Bolivia belong to a new ID genotype. In summary, we identified a new major lineage of enzootic VEEV subtype ID, information that could aid in the understanding of the emergence and evolution of VEEV in South America.

## Author Summary

Venezuelan equine encephalitis virus (VEEV) has been responsible for hundreds of thousands of human and equine cases of severe disease in the Americas. In 2005–2007, cases of Venezuelan equine encephalitis (VEE) were diagnosed for the first time in residents of Bolivia; the patients did not report traveling, suggesting endemic circulation of VEEV in Bolivia. In 2001 and 2003, VEE cases were also identified in Ecuador. We characterize recent VEEV from Bolivia, Ecuador and Peru and compared their relationships to strains from other parts of South America. We found that most VEEV from Peru grouped within a particular genetic lineage known to circulate in Panama and Peru whereas the VEEV circulating in Ecuador belong to a genetic lineage that circulates in Colombia and Venezuela. Importantly, the VEEV from Madre de Dios, Peru and Cochabamba, Bolivia belong to a new genetic lineage. This finding could aid in the understanding of the emergence and evolution of VEEV in South America and underscores the need for continuous monitoring for VEEV activity.

## Introduction

Venezuelan equine encephalitis virus (VEEV), a member of the family *Togaviridae* genus *Alphavirus*, has been responsible for outbreaks involving hundreds-of-thousands of equine and human cases of severe disease in the Americas [1]. At least 14 varieties within the six VEE subtype complex of alphaviruses have been recognized. Only subtype I varieties AB and C have caused major epizootics/epidemics, whereas subtypes II through VI and subtype I varieties D, E and F are enzootic strains that are generally avirulent in horses, but capable of causing human disease [2].

In Ecuador, VEE was first confirmed in 1944 when the virus was isolated from the blood of a sick horse [3]. However, clinical cases that were compatible with VEEV infection had been observed as early as 1940. In 1958, VEEV neutralizing antibodies were found in sera of inhabitants of the Pacific coastal region of Ecuador [4]. In 1968–1969, a large outbreak involving more than 30,000 equids was reported, and in 1975–1977, field work investigations in Ecuador yielded VEEV isolates that were characterized genetically as the Southwestern Colombia/Ecuador ID genotype [5],[6].

In Peru, VEEV was first isolated in the 1940s when subtype IAB caused epizootics and epidemics along the Peruvian coast [7],[8]. Field work investigations were conducted afterwards in the Amazon region of Peru, resulting in the isolation of 11 VEE complex alphavirus strains [9],[10]. Ten isolates were later identified as subtype ID VEEV, whereas one strain was identified as a (new) subtype IIIC virus [9],[10],[11]. Evidence of VEEV human infections was only obtained in 1993–1995, when subtype ID was isolated from febrile patients residing in the Amazon region of Peru [12],[13].

In 2000, in collaboration with the Ministries of Health of Bolivia, Ecuador and Peru, a passive surveillance study was initiated with the purpose of investigating the etiology of febrile illness. As part of the surveillance activities in Peru, several VEEV strains were obtained from febrile patients. Genetic analyses of VEEV strains isolated in Peru prior to 2003 identified two ID circulating genotypes: Colombia/Venezuela and Peru/Panama [14],[15]. In addition, circulation of subtype III variety C and D VEE complex alphaviruses was also detected [14].

Since 2003, VEEV has been continuously isolated from febrile patients living in Iquitos and Yurimaguas (Loreto department) and more recently from patients living in Puerto Maldonado, (Madre de Dios department) in Peru. During these surveillance activities, two strains of VEEV were also isolated from an area in Ecuador where VEE had been not previously reported. In addition, from 2005–2007, VEEV was isolated from febrile residents of Eterazama in Cochabamba, Bolivia, providing, for the first time, evidence that VEEV also circulates in this country. In this study, we sought to investigate the genetic relationship among the VEEV strains isolated in Bolivia, Ecuador and Peru, and compare them to isolates from other countries in South America.

## Materials and Methods

### Study subjects

The study protocols were approved by the Ministry of Health of the participant countries and the Naval Medical Research Center Institutional Review Board (protocols NMRCD.2000.0006, NMRCD.2001.0002, NMRCD.2000.0008) in compliance with all applicable Federal regulations governing the protection of human subjects. The study subjects were patients who presented with a diagnosis of acute, febrile undifferentiated illness in their home or at military or civilian outpatient clinics at the specimen collection study sites described below. A signed consent form was obtained from each volunteer after they were informed about the study and a standardized questionnaire was used to obtain demographic and clinical information from each patient at the time of voluntary enrollment. Travel history information was also recorded. The criteria for inclusion in the program have been described previously and consist of fever 38°C or higher and no more than 7 days duration accompanied by headache, myalgia, or other nonspecific symptoms such as ocular and/or joint pain, generalized fatigue, cough, nausea, vomiting, sore throat, rhinnorhea, difficulty breathing, diarrhea, bloody stool, jaundice, dizziness, disorientation, stiff neck, petecchiae, ecchymoses, bleeding gums or nose [12],[13]. During the acute phase of illness blood samples were obtained from each patient, and when possible, convalescent samples were obtained 10 days to 4 weeks later for serological studies.

### Study sites

Human VEEV isolates included in this study were obtained from the specimen collection sites in Peru located in the city or around Iquitos and Yurimaguas in the department of Loreto and Puerto Maldonado in Madre de Dios. Iquitos is a city of about 400,000 residents located in the Amazon River basin in the Department of Loreto approximately 120 meters above sea level. Yurimaguas is located at the confluence of the Huallaga and Paranapura rivers in the steamy rainforests of northeastern Peru with a population of 63,000 habitants.

Puerto Maldonado is one of the most important cities of the southern jungle. It sits on the banks of the Madre de Dios river, which connects it with Rivera Alto in Bolivia and with Assis, in Brazil (242 km). Puerto Maldonado is a city of approximately 25,000 habitants and is located about 256 meters above sea level.

VEEV isolates were also obtained from Eterazama, Cochabamba department in Bolivia. Eterazama is located approximately 450 meters above sea level. At the time of the 2001 census, it had a population of about 2,500 people.

**Figure 1:**
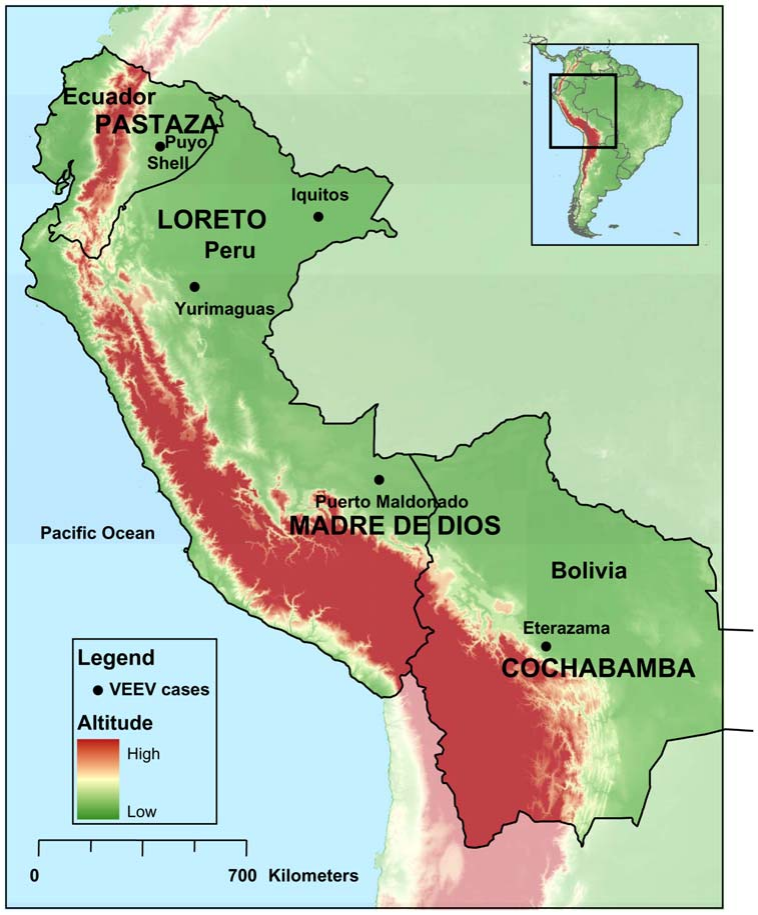
Geographic distribution of the sites in Ecuador, Peru, and Bolivia with confirmed cases of VEE.

Two VEEV strains were obtained from Shell and Puyo located in the Pastaza province of the Amazon River basin of Ecuador. Puyo is located approximately 950 meters above sea level and in 2006 it had a population of about 25,000 inhabitants. Shell is a town of 5,000 people located 5 km from Puyo. Figure 1 shows the geographic distribution of the sites with confirmed VEE cases identified as part of this febrile surveillance study.

### Virus isolation

Patient specimens were sent to the biosafety level 3 containment laboratory at NMRCD. Sera were diluted 1∶10 in Eagle's minimum essential medium (EMEM) supplemented with 2% fetal bovine serum, 200 µg of streptomycin, and 200 U/ml of penicillin. Diluted sera were then inoculated into monolayers of confluent African green monkey kidney epithelial cells (Vero) and *Aedes albopictus* mosquito (C6/36) cells. Slides were prepared from the infected cells and an immunofluorescence assay (IFA) was performed using polyclonal antibodies against several arboviruses endemic in Peru [12],[13],[14],[15],[16],[17]. A variety of arboviruses such as dengue, Oropouche, Mayaro, group C, yellow fever and guaroa were isolated from these samples, including the human VEEV isolates listed in Table 1 that were selected for sequencing.

**Table 1:** List of VEE isolates included in the study.

Strain	Location (Town, department, Country)	Month/year	Age	Gender
OBT 4572	Eterazama, Cochabamba, Bolivia	April 2005	56	Male
OBT 4574	Eterazama, Cochabamba, Bolivia	April 2005	18	Male
OBT 4581	Eterazama, Cochabamba, Bolivia	April 2005	51	Female
FVB 200	Eterazama, Cochabamba, Bolivia	March 2006	29	Male
FVB 204	Eterazama, Cochabamba, Bolivia	April 2006	21	Female
FVB 258	Eterazama, Cochabamba, Bolivia	February 2007	23	Female
FSE 507	Puyo, Pastaza, Ecuador	May 2001	30	Male
FSE 429	Shell, Pastaza, Ecuador	June 2003	12	Male
IQD 3758	Iquitos, Loreto, Peru	September 2002	19	Male
FSL 985	Iquitos, Loreto, Peru	August 2003	30	Female
FSL 995	Iquitos, Loreto, Peru	September 2003	20	Male
FSL 1063	Iquitos, Loreto, Peru	November 2003	23	Female
FSL 1065	Iquitos, Loreto, Peru	November 2003	24	Male
IQD 8361	Iquitos, Loreto, Peru	October 2004	30	Male
IQD 9923	Iquitos, Loreto, Peru	January 2005	42	Female
NFI 144	Iquitos, Loreto, Peru	January 2005	34	Female
IQE 1568	Iquitos, Loreto, Peru	June 2005	15	Male
NFI 276	Iquitos, Loreto, Peru	February 2006	28	Male
IQE 2879	Iquitos, Loreto, Peru	February 2006	15	Female
IQE 3485	Iquitos, Loreto, Peru	March 2006	19	Female
IQE 3755	Iquitos, Loreto, Peru	April 2006	25	Male
IQE 3963	Iquitos, Loreto, Peru	May 2006	12	Female
NFI 413	Iquitos, Loreto, Peru	June 2006	18	Male
IQE 4129	Iquitos, Loreto, Peru	July 2006	11	Female
IQE 4267	Iquitos, Loreto, Peru	August 2006	71	Male
IQE 5234	Iquitos, Loreto, Peru	April 2007	8	Female
IQE 5244	Iquitos, Loreto, Peru	April 2007	33	Male
IDA 85	Iquitos, Loreto, Peru	June 2007	12	Male
FSL 1137	Yurimaguas, Loreto, Peru	January 2004	37	Male
OBT 4458	Yurimaguas, Loreto, Peru	January 2006	49	Male
FSL 2314	Yurimaguas, Loreto, Peru	January 2006	3	Male
FSL 2649	Yurimaguas, Loreto, Peru	July 2006	32	Male
FMD 320	Puerto Maldonado, Madre de Dios, Peru	March 2005	39	Male
FMD 749	Puerto Maldonado, Madre de Dios, Peru	January 2006	18	Male
FMD 1017	Puerto Maldonado, Madre de Dios, Peru	February 2007	66	Female
FMD 1070	Puerto Maldonado, Madre de Dios, Peru	February 2007	11	Male
FMD 1737	Puerto Maldonado, Madre de Dios, Peru	December 2007	18	Male
FMD 1905	Puerto Maldonado, Madre de Dios, Peru	February 2008	21	Female

### RNA extraction, RT, and PCR amplification

Viral RNA was extracted using the QIAamp viral RNA mini kit (Qiagen, Valencia, CA) or Trizol reagent (Invitrogen, Carlsbad, California) following the manufacturer's protocols. The reverse transcription (RT) reaction was done using 1× RT buffer, 0.2 mM dNTPs, 1 µM of primers, 80 U of RNAsin ribonuclease inhibitor (Promega, Madison, WI), 1 mM of dithiothreitol (DTT), 200 U of SuperScript reverse transcriptase (Invitrogen), and 5 µl (1/10^th^) of the extracted RNA. The reactions were incubated at 42°C for 1 hr. The polymerase chain reaction (PCR) included 1× PCR buffer, 0.2 mM dNTPs, 1 µM of primers, 3 mM of MgCl_2_, 2.5 U of GoTaq DNA polymerase (Promega, Madison, WI) and 20% of RNA. The conditions for the PCRs included incubation at 95°C for 2 min, 35 cycles of 95°C for 30 sec, 48°C for 30 sec, 72°C for 1 min. A final extension of 72°C for 10 min was used to ensure complete double-stranded DNA synthesis. The primers used for the PCR amplification and sequencing reaction have been previously described [14],[18].

### Sequencing and phylogenetic analyses

Purified PCR products were sequenced directly, and sequencing analyses of the PCR products was performed using an Applied Biosystems (Foster City, CA) Prism automated DNA sequencing kit according to the manufacturer's protocol. Deduced amino acid sequences were aligned using the ClustalW algorithm in the MacVector version 9.0 software package (MacVector, Inc., Cary, NC), and the nucleotide sequences were aligned manually based on codon positional homology and compared to VEEV sequences from previously published studies available in the genbank database [5],[14],[15],[19]. Phylogenetic analyses were performed using the neighbor joining, maximum parsimony, and maximum likelihood algorithms implemented in the PAUP* version 4.0 software package [20],[21]. The outgroup consisted of homologous sequences of 4 major lineages of eastern equine encephalitis virus (EEEV). For the neighbor joining analysis, the HKY85 distance formula was used, and bootstrap analyses [22] were performed with 1,000 replicates to place confidence values on the nodes within trees. For the maximum parsimony analysis, the heuristic algorithm was employed.

For maximum likelihood analysis, the general time-reversible (GTR) model of nucleotide substitution was used, with a proportion of 0.255 nucleotide sites being invariable and a gamma distribution among-site rate variation (alpha shape parameter) of 0.726. The starting tree in the analysis was found using neighbor joining, which was followed by successive rounds of tree bisection reconstruction branch-swapping, identifying the maximum likelihood substitutions parameter at each stage until the tree of highest likelihood was found. Bootstrapping was subsequently performed to assess the robustness of tree topologies using 1,000 replicate neighbor joining trees under a maximum likelihood substitution model.

### Antigenic characterization

To evaluate antigenic differences between the VEEV subtype ID Panama/Peru and Peru/Bolivia genotypes, convalescent sera from patients from Madre de Dios (infected with subtype ID Peru/Bolivia genotype) and Loreto, Peru (infected with subtype ID Panama/Peru genotype) and Cochabamba, Bolivia (infected with subtype ID Peru/Bolivia genotype) were tested against homologous and heterologous strains of VEEV. Most convalescent sera were obtained 2 to 4 weeks after infection; however, one sample was collected 7 years after VEE infection.

Samples were processed using a previously described plaque reduction neutralization test (PRNT) [23]. Briefly, sera were heat-inactivated at 56°C for 30 min and 2-fold dilutions were mixed with 100 PFU of virus and incubated at 4°C overnight. The mix was added onto a monolayer of Vero cells and incubated at 37°C for 1 hr before adding an overlay of 0.4% of agarose in EMEM. After 48 hr, plates were stained with 0.25% crystal violet in 20% methanol and plaques were counted. The PRNT titer presented in Table 2 is the reciprocal of the highest serum dilution capable of neutralizing 80% of approximately 100 plaque-forming units (PFU) of virus. The traditional serological criteria was used to determine whether the viruses were antigenically distinguishable (i.e. at least fourfold difference between the homologous and heterologous titers of both, or one but not both of the two sera tested) [24].

**Table 2:** Antigenic characterization of the isolates.

	Virus strain (ID genotype)		
Convalescent sera (ID genotype)	OBT4581 (Peru/Bolivia)	FSL201 (Panama/Peru)	FSL205 (Panama/Peru)
FMD1278 (Peru/Bolivia)	320	320	320
FVB161 (Peru/Bolivia)	**160**	160	320
FMD657 (Peru/Bolivia)	20	40	40
FMD1789 (Peru/Bolivia)	640	640	640
FSL202 (Panama/Peru)	≤20	**20**	≤20
FSL206 (Panama/Peru)	320	640	**320**
EMB 4544(*)	640	ND	640

Homologous titers (reciprocal virus isolate vs convalescent sera obtained from the same patient) are represented in bold.

*: Convalescent sera obtained 7 years after VEE infection.

ND, not done.

## Results

### Distribution and description of VEE cases

Human cases of VEE have been previously described in Iquitos [12],[13],[14],[19] and more recently in Yurimaguas [25]. However, in 2005, cases of VEE were reported for the first time in Puerto Maldonado, Madre de Dios. Overall, between 2003–2008 VEEV was isolated from 86 febrile patients in Iquitos, 11 patients in Yurimaguas and 8 patients in Puerto Maldonado, Peru and the demographic data collected from these recent VEE cases were compared with those obtained prior to 2003 in Peru (n = 50). The main occupations of these patients were students, housewives, farmers, soldiers, teachers among others. A total of 63% of the confirmed cases by virus isolation were males and 37% were females. Most of the patients in Peru with confirmed VEE diagnosis by virus isolation were older than 15 years (n = 155; 83.3%) and only a minority were children (n = 31; 16.7%).

In Ecuador, VEEV was isolated from Shell and Puyo in the Pastaza province, areas with no prior reports of VEE. The patients were a physician (a 30 year-old male) and a student (a 12 year-old male). These cases were reported in the Amazon basin region, not far from the border with Peru.

In April 2005, VEEV was isolated for the first time in Cochabamba, Bolivia from a patient with febrile illness. The patient was a 56-year-old male agriculture worker and did not report traveling to known VEE-endemic areas. Subsequent cases were observed in March and April of the same year and in February 2007. None of the patients reported traveling outside of the Cochabamba department.

The nonspecific VEE clinical manifestations among the Bolivian, Ecuadorian and Peruvian patients were very similar and include fever (100%), chills (92%), malaise (97%), headache (84%), hyporexia (79%), myalgia (79%), arthralgia (69%) among others. Throughout the study period, only 3 patients with confirmed VEEV infection (from Bolivia and Peru) developed neurological complications, one of which succumbed to the disease. A detailed description of the fatal case will be described elsewhere. Figure 1 shows the geographical location of the confirmed VEE cases included in this study.

### Genetic characterization of VEEV isolates

**Figure 2:**
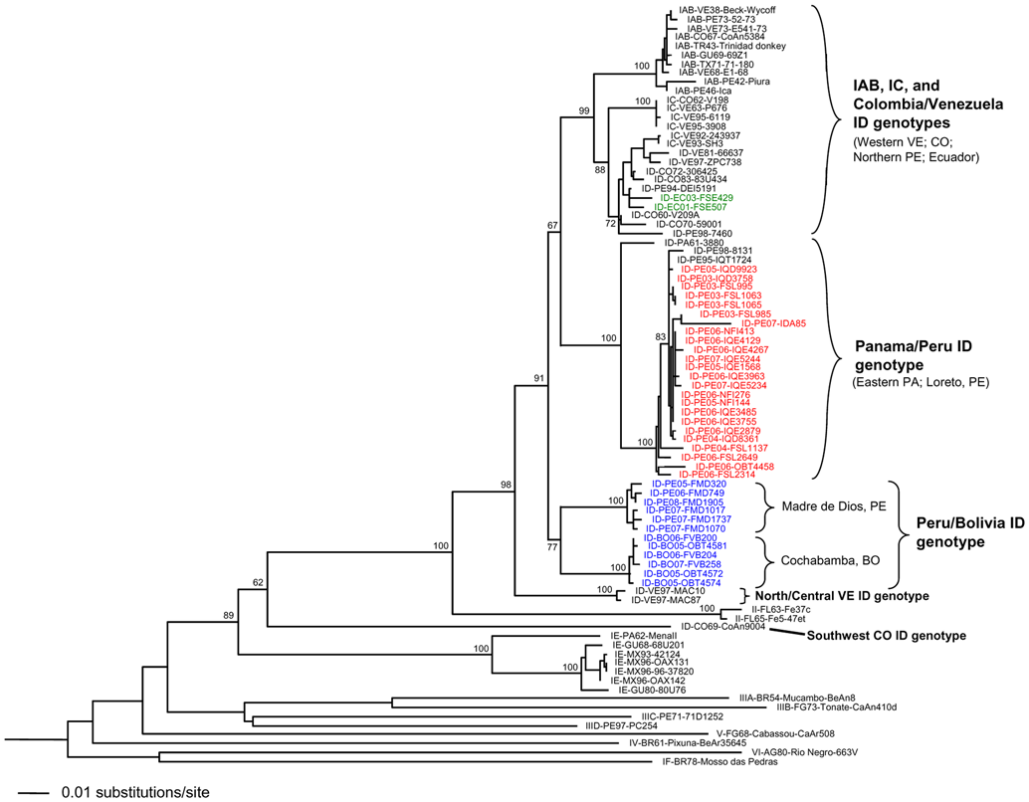
Neighbor joining phylogenetic tree of the Venezuelan equine encephalitis virus (VEEV) complex. The tree was derived from partial envelope glycoprotein precursor (PE2) gene sequences of recent VEEV isolates from Ecuador (green), Peru (red), and Bolivia (blue) and previously published homologous sequences, using the neighbor joining program implemented in PAUP* version 4.0 [31]. The tree was rooted using an outgroup comprised of 4 major lineages of eastern equine encephalitis virus [32]; the outgroup has been removed to improve resolution of the figure. Virus strains are labeled by VEE complex subtype, abbreviated country name and year of isolation (last two digits of year only), followed by the strain or code designation. The abbreviated country names are as follows: PA, Panama; GU, Guatemala; MX, Mexico; FL, Florida; TX, Texas; TR, Trinidad; FG, French Guiana; VE, Venezuela; CO, Colombia; BR, Brazil; AG, Argentina; PE, Peru; EC, Ecuador; BO, Bolivia. Numbers indicate bootstrap values for the clades to the right. Trees generated using maximum parsimony and maximum likelihood methods had identical topologies except for some groupings between subtypes III and V.

In order to determine the genetic relationship among the new VEEV isolates from Bolivia, Ecuador, and Peru and the strains from other areas in South America including Colombia and Venezuela, RT-PCR amplifications and sequencing of the partial PE2 gene (815 bp) were carried out. This genome region was chosen because there is an extensive GenBank database of sequences available for comparison, and because this area undergoes critical amino acid substitutions that are associated with VEE epizootic/epidemic emergence [14],[15],[18],[26],[27]. Recent VEEV strains were selected for sequencing based on date of collection and geographic origin. Overall, the phylogenetic trees that were generated in this study, using neighbor joining, maximum parsimony, and maximum likelihood methods, had identical topologies except for some groupings between subtypes III and V (Figure 2). Twenty-four isolates from Peru grouped with the Panama/Peru ID genotype with strong bootstrap support, and no evidence of circulation of the Colombia/Venezuela ID genotype in Peru was observed among the recent Peruvian isolates.

Interestingly, the 12 strains from Puerto Maldonado, Madre de Dios in Peru and Eterazama (Cochabamba) in Bolivia formed a distinct clade within subtype ID that is a sister to the Colombia/Venezuela and the Peru/Panama genotypes. Sequence comparisons between the Panama/Peru and Bolivia/Peru ID genotype viruses revealed about a 3% difference at the nucleotide level and a 0.4% difference at the amino acid level whereas the Colombia/Venezuela and Peru/Panama genotypes are known to differ by about 5% at the nucleotide level and 0.8% at the amino acid level [14]. A 1.7% nucleotide difference was observed between the Colombia/Venezuela and Peru/Bolivia genotypes.

The two Ecuadorian strains that were obtained from patients in 2001 and 2003 grouped within the Colombia/Venezuela ID genotype, which also includes the epidemic/epizootic subtype IAB and IC strains. Previous isolates of ID genotype viruses from Ecuador have grouped with the Southwest Colombia/Ecuador ID genotype [5]. However, these isolates were collected on the western side of the Andes mountains, while the two new isolates were collected from the eastern side of this mountain range, in the Amazon basin.

### Antigenic characterization of the Panama/Peru and Peru/Bolivia ID genotypes

Although the Panama/Peru and the new Bolivia/Peru ID genotypes differ by only 3% at the nucleotide level, we examined the possibility that they are antigenically distinguishable. To test this possibility, convalescent sera from patients infected with either the Panama/Peru ID genotype or the new Bolivia/Peru ID genotype strain were tested for their ability to neutralize homologous and heterologous viruses. The results demonstrated that the convalescent sera from these patients equally neutralize both genotype strains (Table 2), suggesting that these strains are not antigenically different. Because the convalescent sera were obtained 2 weeks to a month after infection, we also included in the analyses serum from a volunteer who contracted VEE infection (with the Panama/Peru genotype strain) 7 years before. The results obtained with this serum also failed to antigenically discriminate between the Panama/Peru vs the Peru/Bolivia genotypes. Overall, the VEE neutralizing titers range between 20 to 640 (Table 2).

The lack of convalescent sera from the Colombia/Venezuela genotype strains prevented us from performing similar testing. We did not attempt to produce antibodies in animals to test antigenic differences.

## Discussion

VEEV continues to cause sporadic outbreaks of severe febrile disease in South America. In 2005, cases of VEE were detected for the first time in Eterazama, Cochabamba department in Bolivia. Prior to this report, there was no evidence of VEEV circulation in this country, and more importantly, there was no proof that VEEV was responsible for human illness in Bolivia. The continuous isolation of VEEV from Bolivian (2005–2007) patients with no report of traveling suggests endemic circulation of the virus in Cochabamba. Because our surveillance activities in Cochabamba, Bolivia were initiated only in 2005, it is difficult to assess how long the virus has been present in this area. In addition, the limited extent of our surveillance activities in Bolivia that includes only the Beni, Cochabamba and Santa Cruz departments may be preventing us from detecting VEEV cases in other areas in Bolivia.

Surveillance activities in Madre de Dios, Peru began in 2004 but it was not until 2005 that the first VEE human cases were reported. The construction of the Interoceanic Highway that began in 2000 and is scheduled for completion in 2010 has disrupted the ecology in Madre de Dios (where most of the construction activities are currently undergoing) and cause an increase in forest disturbance [28]. The highway, which creates a coast to coast trucking route between the coastal cities of Ilo, Matarani and Marcona in Peru and the Brazilian ports of Rio de Janeiro and Santos, is therefore possibly causing the emergence of new viruses in the area. In addition to the highway, gold mining extraction practices have also intensified during recent years, which is probably another key factor for the emergence of VEE and other arboviruses in Madre de Dios. Only between 2000 and 2005, Peru had the world sixth highest loss of old-growth forests, losing 224,600 hectares per year [28].

In Ecuador, VEEV was first detected in 1944 when the virus was isolated from the blood of a sick horse [3]. In 1968–1969, a large outbreak involving more than 30,000 equines was reported in Ecuador, which later extended to Central America, Mexico and the United States [6],[29],[30]. In 1975–1977, ecological activities in Ecuador yielded VEEV isolates that were genetically characterized as the Southwestern Colombia/Ecuador ID genotype [5]. In our study, surveillance activities in Ecuador yielded the isolation of two VEEV subtype ID strains from febrile patients residing in the Pastaza province, areas with previously unknown VEE activity. Phylogenetic analyses revealed that these new isolates group within the Colombia/Venezuela genotype, which apparently gives rise periodically to the epidemic/epizootic IAB and IC strains [14],[15],[18],[26],[27]. Due to the fact that the new isolates came from a different geographic region (east of the Andes Mountains) than the older Ecuadorian isolates from coastal regions makes it difficult to assess whether the Southwestern Colombia/Ecuador ID genotype is still circulating in Ecuador.

In Peru, VEEV subtype IAB was the cause of equine epizootics along the Pacific coast in the 1940s, 1950s, 1969 and 1973 [7]. No other epizootic has been reported since; however, enzootic VEE complex alphaviruses have been continuously isolated from mosquitoes, rodents, and humans. Most of these isolates were obtained from Iquitos, which is an endemic area of VEEV circulation [14],[17]. Previous genetic analyses of the VEEV strains from Peru identified two distinct ID genotypes responsible for human illness: Colombia/Venezuela and Panama/Peru. In addition, isolations of VEE complex subtype IIIC and IIID were also reported; the latter being responsible for human illness in Iquitos similar to subtype ID VEEV [14].

In this study, we sought to determine the genetic relationship of the VEEV Peruvian strains isolated after 2003 and to determine the genetic relationships of the new Ecuadorian and Bolivian strains of VEEV to strains previously isolated in South America. Phylogenetic analyses revealed that the strains from Bolivia form a new clade that also includes the strains from Madre de Dios, Peru. This new clade is a sister to the Colombia/Venezuela and Panama/Peru genotypes and related genetically to the North/Central Venezuelan VEEV. More detailed complete sequence analyses of the newly recognized Bolivian-Peru genotype viruses and genetic comparison with IAB and IC viruses is needed to identify potential mutations critical for the emergence of epizootic viruses.

In summary, this study has identified a new genetic lineage of VEE subtype ID, the Bolivia-Peru ID genotype. This information could aid in the understanding of the emergence and evolution of VEEV in South America. Further ecological and surveillance activities are needed in Madre de Dios, Peru and Bolivia to identify the vectors and reservoir host(s) involved in transmission and to determine the public health impact and distribution of VEEV in the region. In addition, field investigations are needed to examine the possibility of emergence of epizootic strains of VEEV in these areas.

## Supporting Information

Alternative Language Abstract S1 Translation of the abstract into Spanish by PVA.
(0.03 MB DOC)

## References

[1] Weaver SC, Salas R, Rico-Hesse R, Ludwig GV, Oberste MS (1996). Re-emergence of epidemic Venezuelan equine encephalomyelitis in South America. VEE Study Group.. Lancet.

[2] Weaver SC, Ferro C, Barrera R, Boshell J, Navarro JC (2004). Venezuelan equine encephalitis.. Annu Rev Entomol.

[3] Sotomayor C (1946). A study of the virus of equine encephalomyelitis in Ecuador.. J Am Vet Med Assoc.

[4] Baquerizo Amador L, Marmol F (1958). [Viral encephalitis transmitted by arthropods. IV. Investigation of the Venezuelan type in some human blood on the Ecuadorian coast.].. Rev Ecuat Hig Med Trop.

[5] Powers AM, Oberste MS, Brault AC, Rico-Hesse R, Schmura SM (1997). Repeated emergence of epidemic/epizootic Venezuelan equine encephalitis from a single genotype of enzootic subtype ID virus.. J Virol.

[6] Gutierrez E, Monath TP, Alava A, Uriguen D, Arzube M, Chamberlain RW (1975). Epidemiologic investigations of the 1969 epidemic of Venezuelan encephalitis in Ecuador.. American Journal of Epidemiology.

[7] Johnson KM, Martin DH (1974). Venezuelan equine encephalitis.. Adv Vet Sci Comp Med.

[8] Walton TE, Grayson MA, Monath TP (1988). Venezuelan equine encephalitis.. The arboviruses: epidemiology and ecology.

[9] Scherer WF, Madalengoitia J, Flores W, Acosta M (1975). The first isolations of eastern encephalitis, group C, and Guama group arboviruses from the Peruvian Amazon region of western South America.. Bull Pan Am Health Organ.

[10] Scherer WF, Anderson K (1975). Antigenic and biologic characteristics of Venezuelan encephalitis virus strains including a possible new subtype, isolated from the Amazon region of Peru in 1971.. Am J Epidemiol.

[11] Scherer WF, Chin J (1983). An unusual strain of Venezuelan encephalitis virus existing sympatrically with subtype I-D strains in a Peruvian rain forest.. Am J Trop Med Hyg.

[12] Watts DM, Callahan J, Rossi C, Oberste MS, Roehrig JT (1998). Venezuelan equine encephalitis febrile cases among humans in the Peruvian Amazon River region.. Am J Trop Med Hyg.

[13] Watts DM, Lavera V, Callahan J, Rossi C, Oberste MS (1997). Venezuelan equine encephalitis and Oropouche virus infections among Peruvian army troops in the Amazon region of Peru.. Am J Trop Med Hyg.

[14] Aguilar PV, Greene IP, Coffey LL, Medina G, Moncayo AC (2004). Endemic Venezuelan equine encephalitis in northern Peru.. Emerg Infect Dis.

[15] Oberste MS, Weaver SC, Watts DM, Smith JF (1998). Identification and genetic analysis of Panama-genotype Venezuelan equine encephalitis virus subtype ID in Peru.. Am J Trop Med Hyg.

[16] Travassos da Rosa AP, Turell MJ, Watts DM, Powers AM, Vasconcelos PF (2001). Trocara virus: a newly recognized Alphavirus (Togaviridae) isolated from mosquitoes in the Amazon Basin.. Am J Trop Med Hyg.

[17] Turell MJ, O'Guinn ML, Jones JW, Sardelis MR, Dohm DJ (2005). Isolation of viruses from mosquitoes (Diptera: Culicidae) collected in the Amazon Basin region of Peru.. J Med Entomol.

[18] Moncayo AC, Medina GM, Kalvatchev Z, Brault AC, Barrera R (2001). Genetic diversity and relationships among Venezuelan equine encephalitis virus field isolates from Colombia and Venezuela.. Am J Trop Med Hyg.

[19] Morrison AC, Forshey BM, Notyce D, Astete H, Lopez V (2008). Venezuelan equine encephalitis virus in iquitos, peru: urban transmission of a sylvatic strain.. PLoS Negl Trop Dis.

[20] Swofford DL, Associates SMS (1998). Phylogenetic analysis using parsimony (*and other methods).. 4 ed.

[21] Wilgenbusch JC, Swofford D (2003). Inferring evolutionary trees with PAUP*.. Curr Protoc Bioinformatics Chapter.

[22] Felsenstein J (1985). Confidence limits on phylogenies: An approach using the bootstrap.. Evolution.

[23] Beaty BJ, Calisher CH, Shope RE, Schmidt NJ, Emmonds RW (1989). Arboviruses.. Diagnostic procedures for viral, rickettsial and chlamydial infections. Sixth ed.

[24] Calisher CH, Karabatsos N, Monath TP (1988). Arbovirus serogroups: definition and geographic distribution.. The Arboviruses: Epidemiology and Ecology.

[25] Vilcarromero S, Laguna-Torres VA, Fernandez C, Gotuzzo E, Suarez L (2009). Venezuelan equine encephalitis and upper gastrointestinal bleeding in child.. Emerg Infect Dis.

[26] Weaver SC, Anishchenko M, Bowen R, Brault AC, Estrada-Franco JG (2004). Genetic determinants of Venezuelan equine encephalitis emergence.. Arch Virol Suppl.

[27] Weaver SC, Pfeffer M, Marriott K, Kang W, Kinney RM (1999). Genetic evidence for the origins of Venezuelan equine encephalitis virus subtype IAB outbreaks.. Am J Trop Med Hyg.

[28] Oliveira PJ, Asner GP, Knapp DE, Almeyda A, Galvan-Gildemeister R (2007). Land-use allocation protects the Peruvian Amazon.. Science.

[29] Franck PT, Johnson KM (1971). An outbreak of Venezuelan equine encephalomeylitis in Central America. Evidence for exogenous source of a virulent virus subtype.. Am J Epidemiol.

[30] Zehmer RB, Dean PB, Sudia WD, Calisher CH, Sather GE (1974). Venezuelan equine encephalitis epidemic in Texas, 1971.. Health Serv Rep.

[31] Swofford DL (1998). PAUP* Phylogenetic analysis using parsimony (* and other methods).

[32] Brault AC, Powers AM, Chavez CL, Lopez RN, Cachon MF (1999). Genetic and antigenic diversity among eastern equine encephalitis viruses from North, Central, and South America.. Am J Trop Med Hyg.

