# Supplementary material for: Genetic Characterization of Venezuelan Equine Encephalitis Virus from Bolivia, Ecuador and Peru: Identification of a New Subtype ID Lineage
Source: PLoS Negl Trop Dis. 2009 Sep 15;3(9):e514. doi: 10.1371/journal.pntd.0000514 (PMC2734058; doi:10.1371/journal.pntd.0000514)
Supplement: Alternative Language Abstract S1 — Translation of the abstract into Spanish by PVA. (0.03 MB DOC) [file pntd.0000514.s001.doc]

RESUMEN

El virus de la encefalitis equina venezolana (EEV) ha sido responsable de cientos de miles de casos de enfermedad severa en humanos y caballos en América. Se inició un estudio de vigilancia pasiva en Perú, Bolivia y Ecuador para determinar los agentes etiológicos responsables de enfermedad febril. En el estudio se admitieron pacientes con enfermedad febril en fase aguda (<7 días de duración) con sospecha de ser causada por un agente viral y se recolectaron muestras de sangre que fueron analizadas por aislamiento viral. Los datos clínicos y demográficos de cada paciente fueron colectados en el momento de la admisión voluntaria al protocolo. Durante el año 2005 al 2007, se diagnosticaron casos de encefalitis equina venezolana por primera vez en residentes de Bolivia; los pacientes no reportaban viajes a zonas endémicas de circulación de EEV, sugiriendo circulación endémica de EEV en Bolivia. En el 2001 y 2003, también se identificaron casos de EEV en Ecuador. Desde 1993, continuamente se ha aislado el EEV de pacientes en Loreto, Perú y recientemente (2005) en Madre de Dios, Perú. Análisis filogenéticos fueron realizados con aislamientos de EEV de Bolivia, Ecuador y Perú y aislamientos de otros países de Sudamérica. Dentro del subtipo ID de EEV, el genotipo Panamá/Perú fue el predominante en el Perú. Los nuevos aislamientos de Ecuador se agruparon dentro del genotipo Colombia/Venezuela y los aislamientos de EEV de Madre de Dios, Perú y Cochabamba, Bolivia formaron un nuevo genotipo dentro del subtipo ID. En resumen, se identificó un nuevo linaje dentro del subtipo ID del EEV, información que puede ser de suma importancia para entender la emergencia y evolución del EEV en Sudamérica.
